# The Burden of Human Bocavirus 1 in Hospitalized Children With Respiratory Tract Infections

**DOI:** 10.1093/jpids/piad027

**Published:** 2023-04-26

**Authors:** Hedda Trømborg Jalving, Inger Heimdal, Jonas Valand, Kari Risnes, Sidsel Krokstad, Svein Arne Nordbø, Henrik Døllner, Andreas Christensen

**Affiliations:** Department of Clinical and Molecular Medicine, Norwegian University of Science and Technology (NTNU), Trondheim, Norway; Department of Clinical and Molecular Medicine, Norwegian University of Science and Technology (NTNU), Trondheim, Norway; Department of Clinical and Molecular Medicine, Norwegian University of Science and Technology (NTNU), Trondheim, Norway; Department of Clinical and Molecular Medicine, Norwegian University of Science and Technology (NTNU), Trondheim, Norway; Children’s Department, St. Olavs Hospital, Trondheim University Hospital, Trondheim, Norway; Department of Medical Microbiology, St. Olavs Hospital, Trondheim University Hospital, Trondheim, Norway; Department of Clinical and Molecular Medicine, Norwegian University of Science and Technology (NTNU), Trondheim, Norway; Department of Medical Microbiology, St. Olavs Hospital, Trondheim University Hospital, Trondheim, Norway; Department of Clinical and Molecular Medicine, Norwegian University of Science and Technology (NTNU), Trondheim, Norway; Children’s Department, St. Olavs Hospital, Trondheim University Hospital, Trondheim, Norway; Department of Clinical and Molecular Medicine, Norwegian University of Science and Technology (NTNU), Trondheim, Norway; Department of Medical Microbiology, St. Olavs Hospital, Trondheim University Hospital, Trondheim, Norway

**Keywords:** children, hospitalization rates, human bocavirus, respiratory tract infections, seasonality

## Abstract

**Background:**

Human bocavirus 1 (HBoV1) is frequently codetected with other viruses, and detected in asymptomatic children. Thus, the burden of HBoV1 respiratory tract infections (RTI) has been unknown. Using HBoV1-mRNA to indicate true HBoV1 RTI, we assessed the burden of HBoV1 in hospitalized children and the impact of viral codetections, compared with respiratory syncytial virus (RSV).

**Methods:**

Over 11 years, we enrolled 4879 children <16 years old admitted with RTI. Nasopharyngeal aspirates were analyzed with polymerase chain reaction for HBoV1-DNA, HBoV1-mRNA, and 19 other pathogens.

**Results:**

HBoV1-mRNA was detected in 2.7% (130/4850) samples, modestly peaking in autumn and winter. Forty-three percent with HBoV1 mRNA were 12–17 months old, and only 5% were <6 months old. A total of 73.8% had viral codetections. It was more likely to detect HBoV1-mRNA if HBoV1-DNA was detected alone (odds ratio [OR]: 3.9, 95% confidence interval [CI]: 1.7–8.9) or with 1 viral codetection (OR: 1.9, 95% CI: 1.1–3.3), compared to ≥2 codetections. Codetection of severe viruses like RSV had lower odds for HBoV1-mRNA (OR: 0.34, 95% CI: 0.19–0.61). The yearly lower RTI hospitalization rate per 1000 children <5 years was 0.7 for HBoV1-mRNA and 8.7 for RSV.

**Conclusions:**

True HBoV1 RTI is most likely when HBoV1-DNA is detected alone, or with 1 codetected virus. Hospitalization due to HBoV1 LRTI is 10–12 times less common than RSV.

## INTRODUCTION

Human bocavirus 1 (HBoV1) is a small DNA virus in the *Parvoviridae* family. HBoV1 was first discovered in nasopharyngeal aspirates (NPA) from children with respiratory tract infections (RTI) in 2005 [1]. HBoV1 has been detected in children with RTI outside hospitals, and in several hospital studies, with a varying prevalence of 2.2%–18.4% [2–8]. Using sensitive polymerase chain reaction (PCR) tests, HBoV1-DNA is frequently detected together with other respiratory viruses. Such viral codetections have been reported in up to 82% of HBoV1-DNA-positive samples [2, 4, 6, 9–12]. HBoV1-DNA is also frequently detected in children without RTI symptoms [2, 9, 10, 13–17]. Hence, it has been difficult to evaluate causality between detection of HBoV1-DNA in NPAs and RTI. Nonetheless, recent studies with different diagnostic approaches provide accumulating evidence of HBoV1 being a true pathogen [2, 13, 18–20]. The frequent detection of HBoV1-DNA in healthy children is proposed to be due to prolonged shedding after acute infection [4, 9, 10, 21].

We have previously shown that HBoV1-mRNA, a marker of an active, replicating virus, was present in only 25% of children with RTI and a positive HBoV1-DNA test, and in none of HBoV1-DNA-positive healthy controls [13]. We and others have proposed the PCR test targeting HBoV1-mRNA to be a better test for a true HBoV1 RTI in children than the standard HBoV1-DNA PCR [12, 13, 19, 22], which has been used in most epidemiological studies. Another limitation in previous epidemiological studies is that most were quite short, with limited ability to assess the seasonal variations of HBoV1 and codetected viruses.

In the present study, our primary objective was to describe the burden of HBoV1 infections in hospitalized children, using HBoV1-mRNA as a marker of replicating HBoV1, as the most valid expression of true HBoV1 infection. For that purpose, we have used an 11-year-long study enrolling children admitted to the hospital with RTI. All children were evaluated clinically, sampled with an NPA, and tested by PCR for HBoV1-DNA, HBoV1-mRNA, respiratory syncytial virus (RSV), and a panel of other respiratory viruses. The prevalence of HBoV1, viral codetection rates, seasonality, as well as the hospitalization incidence rates of HBoV1 infections were calculated and compared to RSV.

## METHODS

### Study Population

Children <16 years old admitted to the Children’s Clinic at St. Olavs Hospital from November 2006 to September 2017 with RTI were prospectively enrolled in the study. Exclusion criteria were cancer, congenital immune defects, treatment with immunomodulating medications, hospital-acquired RTIs, hospital discharge <2 weeks ago, and newborns who had not yet been discharged after delivery.

### Clinical Classification

All children with RTI were examined and treated per hospital routines. Information about current and previous history was collected from caregivers with a standardized form and abstracted by a member of the research group during the hospital stay, or retrieved from medical records after discharge.

The RTI diagnoses were divided into 2 main categories: upper (URTI) and lower RTI (LRTI). URTI was defined as a diagnosis of rhinosinusitis, tonsillitis, pharyngitis, acute laryngitis, and/or otitis media. LRTI included pneumonia, bronchiolitis, bronchitis, and unspecified LRTI. Chronic premorbid conditions included chronic heart disease, neurologic disease, and pulmonary disease. Preterm birth was defined as gestational age <36 weeks.

### Laboratory Methods

We collected NPAs from all included children with RTI at admission (see Supplementary Methods). All samples were analyzed daily at the Department of Medical Microbiology at St. Olavs Hospital. In-house Taq-Man real-time PCR (RT-PCR) was used to detect HBoV1-DNA and 19 other pathogens: human adenovirus (HAdV), human enterovirus (HEV), human coronavirus (HCoV) species OC43, NL63, 229E, and HKU1, human metapneumovirus (HMPV), human parechovirus (HPeV), influenza virus A and B (FluA/B), parainfluenza virus (PIV) types 1–4, RSV, *Bordetella pertussis*, *Chlamydia pneumonia*, and *Mycoplasma pneumoniae*, as described elsewhere [23]. Rhinovirus (RV) was tested using 2 Taq-Man real-time PCR tests (see Supplementary Methods). For all PCR tests, a cycle threshold value (Ct value) <40 was regarded as a positive test. To help ensure specificity for the lowest HBoV1-DNA loads, samples with Ct values 35–40 were retested.

The 302 samples with a positive PCR test for HBoV1 were quantified for HBoV1-DNA viral load and tested for HBoV1-mRNA, as previously described [13]. There was sufficient material for viral load quantification and mRNA testing in 201 and 274 samples, respectively.

We defined group 1 viruses as the more severe viruses RSV, HMPV, PIV types 1–3, or FluA/B, and a group 2 virus as either HAdV, HEV, HCoV, HPeV, RV, or PIV type 4.

### Epidemiological Definitions and Hospital Rate Calculations

We defined the epidemiological year from August of 1 year until July the following year. The 4 seasons, winter, spring, summer, and autumn, were defined as the months of December through February, March through May, June through August, and September through November. Hospitalization rates were calculated based on (1) virological and clinical data from the surveillance study, (2) LRTI diagnoses registered in the hospital’s Patient Administrative System, and (3) population data from Statistics Norway, as previously described [24].

### Statistical Analysis

Categorical data were analyzed with either Pearson *X*^2^ or binary logistic regression, while continuous non-normal distributed data were analyzed with a Mann–Whitney *U*. A significance level of *p* < .05 was considered statistically significant. The strength of associations was reported with odds ratios (ORs) and 95% confidence intervals (CIs). The binary logistic regression analyses were performed using HBoV1-mRNA (positive vs. negative) as the dependent variable, and a variable of interest (number of codetections [0, 1 or ≥2], the codetection of a group 1 virus [yes/no] and the codetection of each individual virus [yes/no]) as the independent variable. All regression analyses were adjusted for age as a continuous variable. In addition, the analysis on the number of codetections was adjusted by the codetection of a group 1 virus (yes/no).

All statistical analyses were performed using IBM SPSS Statistics version 27.0. Illustrations were created in RStudio with the software package ggplot2, and Adobe Illustrator.

### Ethics

Oral and written information was given to the caregiver(s) and children during their hospital stay. Written consent was obtained from caregivers and children >12 years. If a child had not been invited to participate in the study during the hospital stay, invitation letters were sent to the children and their caregivers, with no response after 2 weeks regarded as passive consent.

The study was approved by the Regional Committee for Medical and Health Research Ethics Central in 2006 (No: 4.2006.2289) and 2012 (No: 2012.1042).

## RESULTS

We included 4879 episodes of RTI in hospitalized children <16 years between November 2006 and October 2017 (Supplementary Figure 1). Children with RTI had a median age of 14.9 months, and 60.3% were boys. At least 1 virus was detected in 88.6% of the samples, with the most common being RV (40.7%) and RSV (30.1%), followed by HEV, PIV types 1–4, HCoV, HPeV, HMPV, HBoV1-DNA, HAdV, and FluA/B (Supplementary Table 1).

### Detection of HBoV1-DNA and HBoV1-mRNA

HBoV1-DNA was the eighth most detected virus. Viral codetections were common; 83.1% (251/302) had at least 1 viral codetection, while 42.7% (129/302) had 2 or more.

Of the 274 positive HBoV1-DNA samples available for HBoV1-mRNA analysis, 47.4% (130/274) were positive for mRNA. Thus, the prevalence of HBoV1-mRNA-positive samples in the whole cohort was 2.7% (130/4850). When comparing children with HBoV1-mRNA-positive and -negative samples, the 2 groups were similar concerning baseline data (sex, chronic diseases. and preterm birth), although children with an mRNA-positive sample were slightly younger (Table 1). All HBoV1-mRNA-positive samples had high HBoV1-DNA load (>10^6^ copies/mL), compared to less than half of the HBoV1-mRNA-negative samples (Table 2).

**Table 1.** Comparison of Baseline Characteristics in Children With Respiratory Tract Infection and Detection of HBoV1 DNA, HBoV1-mRNA and RSV

	HBoV1-DNA (*n* = 274)		HBoV1-mRNA+ vs. mRNA-	RSV (*n* = 1447)^a^	HBoV1-mRNA+ vs. RSV
	mRNA+ (*n* = 130)^a^	mRNA- (*n* = 144)	OR (95% CI)		OR (95% CI)
Age, median (interquartile range), months	15.3 (12.1–19.6)	18.3 (13.2–26.9)^b^		7.9 (2.6–18.9)^b^	
Age group (months)					
<6	7 (5.4)	4 (2.8)	Ref.	614 (42.4)	Ref.
6–11	24 (18.5)	23 (16.0)	0.60 (0.1–2.3)	251 (17.3)	8.4 (3.6–19.7)
12–17	56 (43.1)	42 (29.2)	0.76 (0.21–2.8)	196 (13.5)	25.1 (11.2–55.9)
18–23	23 (17.7)	31 (21.5)	0.42 (0.11–1.6)	161 (11.1)	12.5 (5.3–29.7)
24–59	20 (15.4)	43 (29.9)	0.27 (0.07–1.0)	206 (14.2)	8.5 (0.94–2.0)
≥60	0 (0.0)	1 (0.7)	—	19 (1.3)	—
Male sex	85 (65.4)	87 (60.4)	1.2 (0.76–2.0)	838 (57.9)	1.37 (0.94–2.0)
Premature birth (gestational age <36 weeks)	16/120 (13.3)	26/132 (19.7)	0.63 (0.32–1.2)	188/1427 (13.2)	1.0 (0.59–1.8)
≥1 chronic disease^c^	33 (25.4)	43 (29.9)	0.8 (0.47-1.4)	247 (17.1)	1.7 (1.1-2.5)

The data represents the no. (%) of children unless otherwise specified. Strength of associations estimated by Pearson χ^2^ test and binary logistic regression as appropriate, and given as OR and 95% CI. CI, confidence interval; HBoV1, human bocavirus 1; OR, odds ratio; RSV, respiratory syncytial virus.

^a^Twenty children with both HBoV1-mRNA and RSV were included in the HBoV1-mRNA group only.

^b^ *p* < .001 by Mann–Whitney *U* test.

^c^Heart disease, neurologic disease, and/or pulmonary disease.

**Table 2.** Comparison of Virological Findings in Children With Respiratory Tract Infection and the Detection of HBoV1-DNA, by the Presence of HBoV1-mRNA

	HBoV1-DNA (*n* = 274)		
	mRNA+ (*n* = 130)	mRNA− (*n* = 144)	OR (95% CI)
HBoV1-DNA viral load >10^6^ copies/mL	103/103 (100)	43/98 (43.9)	∞ (−)
Number of codetections			
Single HBoV1-DNA detection	34 (26.2)	12 (8.3)	3.9^a^ (1.7–8.9)
1 viral codetection	55 (42.3)	52 (36.1)	1.9^a^ (1.1–3.3)
≥2 viral codetections	41 (31.5)	80 (55.6)	Ref.
Codetection with group 1/group 2 virus			
Codetection with group 1 virus^b^	27/96 (28.1)^c^	66/132 (50.0)^c^	0.34^d^ (0.19–0.61)
Codetection with only group 2 virus^e^	69/96 (71.9)^c^	66/132 (50.0)^c^	Ref.

The data represents the no. (%) of children unless otherwise specified. Strength of associations estimated by Pearson χ^2^ test and binary logistic regression as appropriate, and given as OR and 95% CI. CI, confidence interval; HBoV1, human bocavirus 1; OR, odds ratio; RSV, respiratory syncytial virus.

^a^Adjusted by the detection of severe virus and age as a continuous variable.

^b^Group 1 virus: respiratory syncytial virus, human metapneumovirus, parainfluenza virus types 1–3, and influenza virus A/B.

^c^Among samples with codetection.

^d^Adjusted by age as a continuous variable.

^e^Group 2 virus: All other viruses than those defined as group 1 viruses.

### Viral Codetections in HBoV1-mRNA-Positive and -Negative Samples

HBoV1-mRNA was detected both alone (26.2%), and codetected with 1 (42.3%) or ≥2 (31.5%) other viruses. The corresponding rates in HBoV1-mRNA-negative episodes were 8.3%, 36.1%, and 55.6%, respectively. Samples with single-detected HBoV1-DNA, and samples with HBoV1-DNA codetected with only 1 other virus, were nearly 4 and 2 times more likely of being mRNA-positive, respectively (Table 2). However, the odds for detecting HBoV1-mRNA were lower when HBoV1-DNA was codetected with a group 1 virus (OR 0.34, 95% CI: 0.19–0.61), as compared to samples codetected with only a group 2 virus (Table 2). Similar results were found when we analyzed each codetected virus type separately (Supplementary Table 2). This means that samples with HBoV1-DNA codetected with either RSV or HMPV were less likely to be HBoV1-mRNA-positive than mRNA-negative (Supplementary Table 2). RV appeared in nearly half of all HBoV1-DNA-positive samples and had a border significance with a somewhat lower rate in HBoV1-mRNA-positive samples, whereas other group 2 viruses did not differ (Supplementary Table 2). These data suggest that a true HBoV1 infection, as indicated by the presence of HBoV1-mRNA, is most probable when HBoV1-DNA is detected alone in NPA, but also in some cases, when it is codetected with RV or other group 2 viruses.

### Seasonal Variability

**Figure 1.**
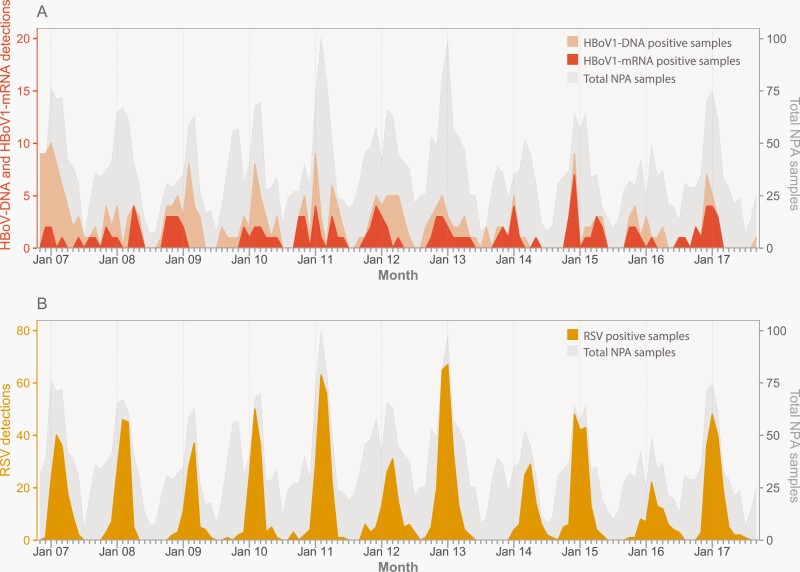
Monthly number of virus detections in children hospitalized with respiratory tract infection at St. Olavs hospital from November 2006 to October 2017. (A) HBoV1-DNA (light orange color) and HBoV1-mRNA detections (dark orange color). (B) RSV detections (yellow color). The gray area represents the total number of NPA samples. HBoV1, human bocavirus 1; NPA, nasopharyngeal aspirate; RSV, respiratory syncytial virus

HBoV1-mRNA was detected in all 11 epidemiological years of the study (Figure 1), with a total number of detections varying between 6 and 17 per year, corresponding to 1.6%–4.1% of all study samples, with a mean of 2.7%.

Both HBoV1-DNA and HBoV1-mRNA had a modest seasonal variation (Figure 1 and Supplementary Figure 2). HBoV1-DNA was most common in the winter and spring seasons, with detections in 7.5% and 6.0% of all samples, appearing simultaneously with large RSV outbreaks. In comparison, HBoV1-mRNA was most common in the autumn (3.1%) and winter (3.4%; Supplementary Figure 2).

### LRTI Hospitalization Rates

Six out of 10 admitted children were hospitalized >24 hours (59.1%, 2882/4875). In total, 6.4% (184/2882) of hospitalized children with LRTI were HBoV1-DNA-positive, and 3.3% were HBoV1-mRNA-positive (95/2870). Children with a positive HBoV1-mRNA test were more likely to acquire an LRTI diagnosis than those with a negative HBoV1-mRNA test (OR 2.9, 95% CI: 1.5–5.6). This difference persisted when adjusting for age and chronic disease (OR 2.9, 95% CI: 1.5–5.8).

The mean yearly rate of children hospitalized >24 hours with LRTI and HBoV1-mRNA was 0.70 per 1000 children <5 years old. The highest hospitalization rate was in the age group 12–23 months, with a mean of 1.9 per 1000 children per year, varying from 0.9 to 4.4 per 1000 children (Supplementary Table 3). Children aged 0–11 months old had a lower yearly mean hospitalization rate of 0.9 per 1000, and children aged 24–59 months had a rate of 0.2 per 1000 children per year.

The mean yearly hospitalization rate of children with an HBoV1-DNA detection was 1.4 per 1000 children <5 years old. Yearly variations are given in Supplementary Table 3.

### Comparison of HBoV1 and RSV

As RSV is a well-known cause of RTI in young children, we compared patients with a true HBoV1 infection, defined as an HBoV1-mRNA-positive sample, to patients infected with RSV. Children with HBoV1-mRNA were older than those with RSV; among <6 months old children only a few percent had HBoV1-mRNA whereas nearly half with RSV belonged to this age group (Table 1). In contrast to RSV, the rate of HBoV1-mRNA detection increased after age 6 months, peaking among children who were 12–17 months old (Table 1). There were no differences in gender or premature birth. Children with chronic disease were more often HBoV1-mRNA-positive than RSV-positive compared with children without chronic disease (Table 1). When adjusting this analysis for age as a categorical variable (<12 months or ≥12 months), ORs in the 2 subgroups (<12 months or ≥ 12 months) differed significantly (*p* for interaction <.001). Children with a chronic disease who were younger than 12 months were more likely to be HBoV1-mRNA-positive than children without a chronic disease (OR 7.2, 95% CI: 3.2–15.9), while children older than 12 months were less likely to be HBoV1-mRNA-positive (OR 0.61, 95% CI: 0.37–1.0).

**Figure 2:**
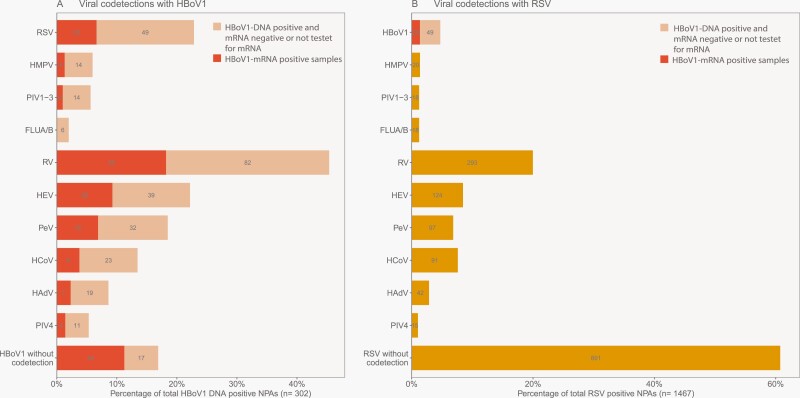
Viral codetections with (A) human bocavirus 1 (HBoV1)-positive nasopharyngeal aspirates (NPAs) and (B) respiratory syncytial virus (RSV)-positive NPAs. Absolute number of codetections with other viruses are listed in the bars. Light orange bar corresponds to HBoV1-DNA-positive samples that are mRNA-negative or not tested for mRNA, and dark orange bar corresponding to HBoV1-positive samples that are mRNA-positive. Combined light and dark orange bars correspond to the total number of HBoV1-DNA-positive samples. The length of the bar corresponds to codetection frequencies in the percentage of all HBoV1 and RSV-positive NPAs, or all HBoV1 or RSV-positive NPAs tested for the relevant virus (missing data in codetections with HBoV1: 26 missing in PeV bar, 64 missing in HCoV bar and 20 missing in PIV4 bar. Missing data in codetections with RSV: 1 missing in HMPV bar, 1 missing in PIV1–3 bar, 49 missing in PeV bar, 270 missing in HCoV bar and 13 missing in PIV4 bar). HMPV, human metapneumovirus; PIV1–3, parainfluenza virus types 1–3; FLU A/B, Influenza virus A and B; RV, rhinovirus; HEV, human enterovirus; PeV, human parechovirus; HCoV, human coronavirus; HAdV, human adenovirus; PIV4, parainfluenza virus type 4.

Compared to RSV, more viral codetections were found in the samples with HBoV1 (Figure 2). The most common codetected viruses with HBoV1-mRNA were RV (42.3%), HEV (21.5%), and RSV (15.4%), while for RSV, the most common codetected viruses were RV (20.0%), HEV (8.5%), and HCoV (7.5%).

While both HBoV1-DNA and HBoV1-mRNA differed significantly by season, they differed less than RSV (Supplementary Figure 2). RSV peaked coinciding with HBoV1-DNA, but after HBoV1-mRNA. In contrast to the low hospitalization rates of HBoV1-mRNA (0.7 per 1000 children <5 years of age), the mean yearly hospitalization rate for children with LRTI and RSV detection was 8.7 per 1000 children <5 years of age, with the highest hospitalization rate in <12-month-old children.

## DISCUSSION

### Main Findings

In this 11-year-long observational study, we detected HBoV1 every year and during all seasons each year. HBoV1-mRNA appeared more often in the autumn and winter. In contrast to RSV, HBoV1-mRNA was seldom detected in children <6 months old, but after 6 months, the detection rate increased and peaked in the 12–17 months age group. Viral codetections were common, appearing in 3 out of 4 children with HBoV1-mRNA. Based on our use of PCR tests for both HBoV1-DNA and HBoV1-mRNA, we suggest that a true HBoV1 infection with active, replicating HBoV1 is likely when HBoV1 is detected as a single virus. However, we also suggest for the first time, that a true HBoV1 infection may be present in some children with codetection of RV or another group 2 virus. On the other hand, the codetections of RSV and other group 1 viruses are less frequently associated with replicating HBoV1. Using HBoV1-mRNA as the most valid marker of true HBoV1 infection, our data show that HBoV1 was likely a cause of hospitalization with LRTI in 0.7 per 1000 children <5 years per year, which was 12 times less than RSV.

### HBoV1-DNA and mRNA Detections

We detected HBoV1-DNA in 6.2% of all RTI episodes, a reliable estimation due to the 11-year-long study period. Data from previous hospital-based studies report detection rates from 2.2% to 18.4% [2–8]. HBoV1-mRNA was only detected in 2.7% of all RTI episodes, which is probably closer to the true rate of HBoV1 infections in hospitalized children. The HBoV1 infection rate is probably higher in the community than in our hospital-based study. In a community-based surveillance study over 1 year in the United States enrolling 28 households, HBoV1 diagnosed by HBoV1-DNA was the second most common virus in all ages and in children <5 years old [16]. Since 2020, the epidemiology of HBoV1 may have changed due to social distancing and other measures during the COVID-19 pandemic, although studies have shown conflicting results [25–28].

### Viral Codetections

Viral codetections were common, appearing in 4 out of 5 children with HBoV1-DNA in our study, in accordance with findings from similar studies [2, 4, 6, 9–11]. Previous research has suggested that this could be related to a long shedding time of HBoV1-DNA up to 12 months after an RTI, and presenting in relation to a new RTI caused by another respiratory virus [17, 29–31]. HBoV1-mRNA-positive samples had fewer codetections than the HBoV1-mRNA-negative samples, and interestingly, there appeared to be a trend between fewer codetections and higher odds of detecting HBoV1-mRNA. Our data suggest that true HBoV1 infection is most probable when HBoV1-DNA is detected alone, or in some cases, when it is codetected with one of the group 2 viruses such as RV and HEV. When HBoV1-DNA was codetected with RSV or other group 1 viruses, fewer were HBoV1-mRNA-positive, indicating that HBoV1 was less likely to contribute to the current infection.

### HBoV1 Seasonality

We show that HBoV1 occurs year-round with a modest seasonal variation. The fact that HBoV1-mRNA was peaking in autumn and winter while HBoV1-DNA peaked later during the year in winter and spring may support our assumption of HBoV1-mRNA being a better indicator of active/early HBoV1 infection whereas HBoV1-DNA may indicate convalescence, but due to the cross-sectional study design, we cannot confirm that. Compared to the pronounced variation of RSV, the seasonal HBoV1 variation was less evident, but the earlier peak of HBoV1-mRNA supports a true seasonality for HBoV1, rather than just a sampling effect during RSV seasons. Previous reports on seasonal variations of HBoV1 adjusting for sampling frequency have been sparse [12]. Famoroti et al. reported a peak of HBoV1-DNA in the summer in South Africa during a study period of almost 5 years [32]. Wu et al. studied HBoV1 in Lanzhou and Nanjing, China, during a period of almost 4 years, and found that HBoV1 was peaking in both December and July [3].

### HBoV1 and Age

The detection rate of HBoV1-mRNA in our hospital population was low in children <6 months old but increased to highest levels in the 12–17 months age group. These findings suggest that infants may be protected from maternal antibodies or some other factors against HBoV1 infection in need of hospitalization, but it might also be related to the fact that most Norwegian children will not be exposed until they start in daycare usually from 1 year of age. However, it has been shown that infants may contract HBoV1 infection [17], so another explanation is that HBoV1 infection in the youngest is a mild disease with limited need of hospitalization. These observations, and in addition to the fact that no children in the present study with HBoV1-infection were readmitted with a new HBoV1-infection (data not shown) support that children may contract a primary HBoV1 infection during the first 6–17 months of life as it has been shown before [17], but we cannot confirm that due to the cross-sectional study design.

### Hospitalization Incidence Rates

The mean yearly hospitalization rate of children hospitalized >24 hours with LRTI and HBoV1-mRNA detection was 0.7 per 1000 children <5 years old during the 11-year-long study period. This was 12 times lower than RSV, and approximately half of the hospitalization rates for endemic HCoV and HMPV previously reported by our group, based on data from the same study (1.5 and 1.9 per 1000 children under 5 years, respectively) [24, 33]. Only 1 study has previously published HBoV1 hospitalization rates. Using the detection of HBoV1-DNA to define HBoV1 infection, Fry et al. studied X-ray-confirmed pneumonia in the Sa Kaeo province in Thailand, and reported a hospitalization rate of 1.23 per 1000 children aged 0–11 months old, and 0.03 per 1000 children aged 1–4 years [34].

### Strengths and Limitations

Although this is a single-center study with a cross-sectional design, it is a strength that it was conducted over 11 consecutive years, and in facilities serving as the sole pediatric hospital for an entire county population of children. This makes the study robust to yearly variations of HBoV1 and viral codetections, thereby making it possible to calculate reliable hospitalization incidence rates. Moreover, the same sampling and in-house PCR test procedures have been used throughout the study period. The results are probably generalizable to other Norwegian hospitals, as well as countries with a similar climate and social structures, but our results should not be interpreted as representative of pediatric populations outside hospitals. The use of the HBoV1-mRNA test is a strength, as we have previously shown it corresponding to clinical infection [13]. Nevertheless, the duration of the HBoV1-mRNA presence in NPAs during infection has not been validated, and we could have missed some potential HBoV1-mRNA-positive samples if the children contacted the hospital in later disease stages. A few children were enrolled with more episodes, and since HBoV1 may shed for a long time [17], there is a risk that these episodes belonged to the same infection. The duration of clinical infection before sampling was not systematically recorded, and therefore not accounted for in the calculations.

## CONCLUSIONS

This study of HBoV1 over an 11-year period shows that HBoV1-DNA was detected in 6.2% of NPAs from children admitted to the hospital with RTI, and approximately half of these have active, replicating viruses (HBoV1-mRNA; 2.7% of all samples). HBoV1-DNA and -mRNA circulates year-round, and exhibits a modest seasonal variation, with HBoV1-mRNA peaking in autumn and winter. Few newborns were hospitalized with HBoV1, but the rate of infected children increased and peaked in the 12–17 months age group. Viral codetections are very common in association with HBoV1. A true HBoV1 infection, as defined by a positive HBoV1-mRNA PCR test result, is most likely in the case of HBoV1-DNA single detection, and when HBoV1-DNA is codetected with RV or another group 2 virus. In contrast, in the presence of RSV and other group 1 viruses, a positive HBoV1-DNA test result is less likely to express a true HBoV1 infection. Our data support that HBoV1 is a relatively uncommon cause of LRTI in children, with a hospitalization rate 12 times lower compared with RSV.

## Supplementary Material

piad027_suppl_Supplementary_File
