# Supplementary material for: The Burden of Human Bocavirus 1 in Hospitalized Children With Respiratory Tract Infections
Source: J Pediatric Infect Dis Soc. 2023 Apr 26;12(5):282–9. doi: 10.1093/jpids/piad027 (PMC10231390; doi:10.1093/jpids/piad027)
Supplement: piad027_suppl_Supplementary_File [file piad027_suppl_supplementary_file.docx]

**Supplementary Material**

Supplementary Methods

Routines for nasopharynx aspirate sampling and testing

Rhinovirus PCR tests

References

Supplementary Figure 1: Flowchart of the study population, number of children admitted to St. Olavs hospital between August 2006 and October 2017 with symptoms of respiratory tract infection (RTI) and sampled with a nasopharyngeal aspirate (NPA) tested for human bocavirus 1 (HBoV1), respiratory syncytial virus (RSV) and 15 other viruses.

Supplementary Table 1: Background and virological data on all RTI episodes included in the study (n=4879).

Supplementary Table 2: Viral codetections in children with respiratory tract infections and HBoV1-DNA in nasopharyngeal samples, by the presence of HBoV1-mRNA, No. (%).

Supplementary Figure 2: Seasonal distribution of A) human bocavirus 1 (HBoV1) DNA and mRNA, and B) respiratory syncytial virus (RSV) in nasopharyngeal aspirate (NPA) samples, as a percentage of all NPA samples each season.

Supplementary Table 3: Yearly hospitalization rates per 1000 children due to lower respiratory tract infection by virus (human bocavirus 1 (HBoV1) DNA, HBoV1-mRNA, and respiratory syncytial virus (RSV)) and age group.

**Supplementary Methods**

**Routines for nasopharynx aspirate sampling and testing**

We collected nasopharynx aspirates (NPAs) from all included children with respiratory tract infections at admission. The NPAs were sampled using a suction catheter put through each nostril and into the nasopharynx, and the aspirate was sampled in a dry tube in a closed system. Antibiotic-free virus medium was subsequently sucked into the tube and the tube was sent to the lab at the Department of Medical Microbiology at St. Olav’s Hospital immediately.

The samples were analyzed daily using in-house PCR-tests and kept cool in a refrigerator until the next day and then kept frozen at -80°C in our biobank established for the project.

**Rhinovirus PCR tests**

From 2006 rhinovirus (RV) was tested using primer pairs and probes targeting genotypes A/B [1]. In 2012 the RV diagnostics was improved by the development of a TaqMan real-time PCR targeting the 5’UTR (untranslated region) part of the virus to include detection of RV C. Primers and probe sequences are shown in the table below. All samples from 2006 up to 2012 were retrospectively analyzed with the new method.

| Table: Primers and probe sequences used to detect rhinovirus C (RV C) | | |
| --- | --- | --- |
| RV_C sense primer | CAGGGTGTGAAGACHCKAGTGTG |  |
| RV_C antisense primer | AACACGGACACCCAAAGTAG |  |
| RV_C_TM | CCCCTGAATGYGGCTAACCTTAAYC | 5'FAM. 3'BHQ1 |

**References**

1. Kristoffersen AW, Nordbø SA, Rognlien AG, Christensen A, Døllner H. Coronavirus causes lower respiratory tract infections less frequently than RSV in hospitalized Norwegian children. Pediatr Infect Dis J **2011**; 30:279-83.


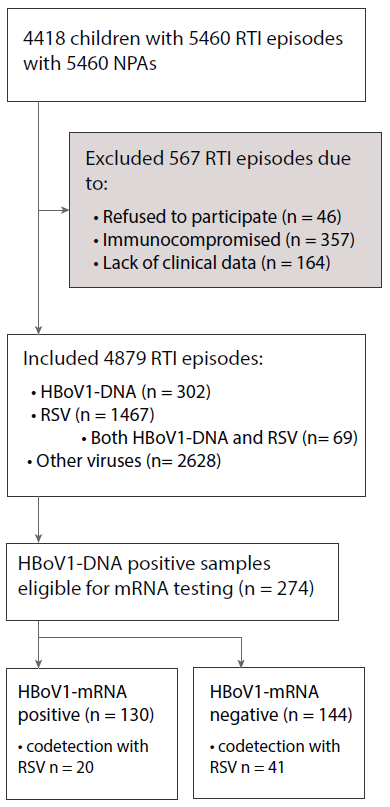
Supplementary Figure 1: Flowchart of the study population, number of children admitted to St. Olavs hospital between August 2006 and October 2017 with symptoms of respiratory tract infection (RTI) and sampled with a nasopharyngeal aspirate (NPA) tested for human bocavirus 1 (HBoV1), respiratory syncytial virus (RSV) and 15 other viruses.

| Supplementary Table 1: Background and virological data on all RTI episodes included in the study (n=4879). | |
| --- | --- |
|  | No. (%) |
| Age, median months (interquartile range) | 14.9 (5.4-27.8) |
| Age groups (months) |  |
| <6 | ()1315 (27.0) |
| 6-11 | 735 (15.1) |
| 12-17 | 806 (16.5) |
| 18-23 | 564 (11.6) |
| 24-59 | 1009 (20.7) |
| ≥60 | 450 (9.2) |
| Male sex | 2941 (60.3) |
| Premature birth | 597/4460 (13.4) |
| ≥1 chronic disease ^a^ | 1168 (23.4) |
| Season^b^ |  |
| Autumn | 1134 (23.2) |
| Winter | 1831 (37.5) |
| Spring | 1344 (27.5) |
| Summer | 570 (11.7) |
| ≥1 viral detection | 4323 (88.6) |
| Detections of group 1 virus |  |
| Respiratory syncytial virus | 1467 (30.1) |
| Human metapneumovirus | 305/4877 (6.3) |
| Parainfluenza virus 1-3 | 358/4875 (7.3) |
| Influenza virus A/B | 235/4877 (4.8) |
| Detection of group 2 viruses |  |
| Rhinovirus | 1986 (40.7) |
| Human enterovirus | 570 (11.7) |
| Human parechovirus | 362 (7.4) |
| Human coronavirus | 336/3959 (8.5) |
| Human adenovirus | 302 (6.2) |
| Parainfluenza virus 4 | 121/4776 (10.2) |
| Data represent No. (%) of children unless otherwise specified. | |
| ^a^Heart disease, neurologic disease, and/or pulmonary disease. | |
| ^a^The seasons autumn, winter, spring and summer are defined as the months September through November, December through February, March through May, and June through August. | |

| Supplementary Table 2: Viral codetections in children with respiratory tract infections and HBoV1-DNA in nasopharyngeal samples, by the presence of HBoV1-mRNA, No. (%). | | | |
| --- | --- | --- | --- |
|  | HBoV1-DNA (n= 274) | |  |
|  | mRNA+ (n= 130) | mRNA- (n= 144) | OR (95% CI) |
| Group 1 viruses |  |  |  |
| Respiratory syncytial virus | 20 (15.4) | 41 (28.5) | 0.38 (0.20-0.71) |
| Human metapneumovirus | 4 (3.1) | 13 (9.0) | 0.30 (0.09-0.97) |
| Parainfluenza virus 1-3 | 3 (2.3) | 11 (7.6) | 0.29 (0.08-1.1) |
| Influenza virus A/B | 0 (0) | 5 (5.5) | 0 (-) |
| Group 2 viruses |  |  |  |
| Rhinovirus | 55 (42.3) | 76 (52.8) | 0.62 (0.38-1.0) |
| Human enterovirus | 28 (21.5) | 36 (25.0) | 0.94 (0.53-1.7) |
| Human parechovirus | 19/129 (14.7) | 31 (21.5) | 0.63 (0.33-1.2) |
| Human coronavirus | 9/105 (8.6) | 18/126 (12.5) | 0.59 (0.25-1.4) |
| Human adenovirus | 7 (5.4) | 17 (11.8) | 0.42 (0.16-1.1) |
| Parainfluenza virus 4 | 4/129 (3.1) | 11 (7.6) | 0.42 (0.13-1.4) |
| Strength of associations estimated by binary logistic regression, adjusted by age as a continuous variable, and given as odds ratio (OR) and 95% confidence intervals (CI). | | | |


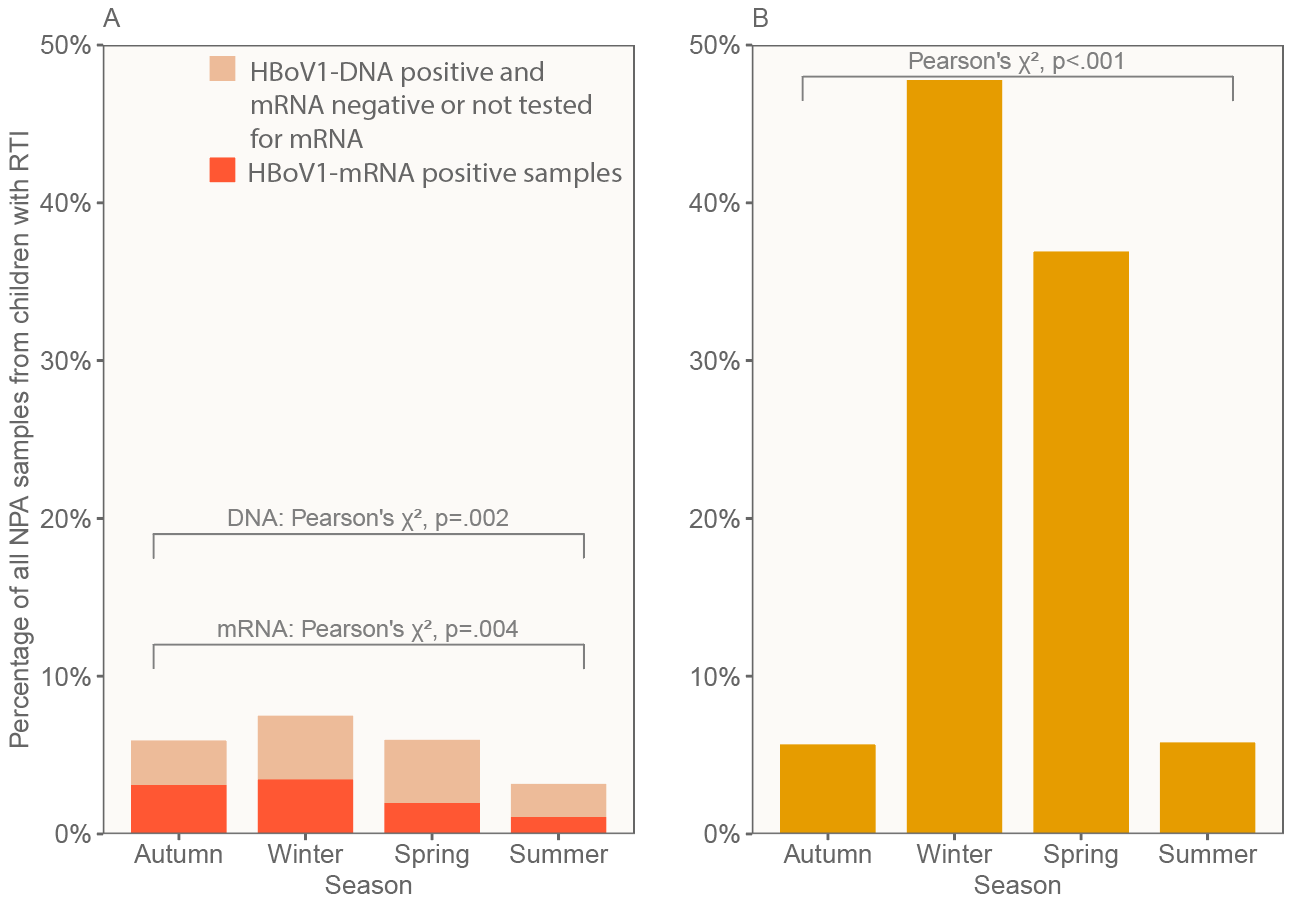


Supplementary Figure 2: Seasonal distribution of A) human bocavirus 1 (HBoV1) DNA and mRNA, and B) respiratory syncytial virus (RSV) in nasopharyngeal aspirate (NPA) samples, as a percentage of all NPA samples each season. In A), light orange bar corresponds to HBoV1-DNA-positive samples that are mRNA-negative or not tested for mRNA, with the dark orange bar corresponding to HBoV1-positive samples that are mRNA-positive. Combined light and dark orange bars correspond to the total number of HBoV1-DNA-positive samples. Autumn is defined as September through November, winter is December through February, spring is March through May and summer is June through August. Brackets represent Pearson χ^2^ test for differences in detections through the different seasons.

| Supplementary Table 3: Yearly hospitalization rates per 1000 children due to lower respiratory tract infection by virus (human bocavirus 1 (HBoV1) DNA, HBoV1-mRNA, and respiratory syncytial virus (RSV)) and age group. | | | | | | | | | | | |
| --- | --- | --- | --- | --- | --- | --- | --- | --- | --- | --- | --- |
|  | **Age 0-11 months** | | |  | **Age 12-23 months** | | |  | **Age 24-59 months** | | |
| **Epidemiological year*** | **HBoV1-DNA** | **HBoV1-mRNA** | **RSV** |  | **HBoV1-DNA** | **HBoV1-mRNA** | **RSV** |  | **HBoV1-DNA** | **HBoV1-mRNA** | **RSV** |
| **2006-2007**** | 3.7 | 0.5 | 30.3 |  | 5.7 | 1.0 | 10.4 |  | 1.6 | 0.4 | 1.9 |
| **2007-2008** | 1.5 | 0.8 | 29.0 |  | 2.7 | 1.6 | 4.7 |  | 0.3 | 0.1 | 1.9 |
| **2008-2009** | 2.2 | 1.3 | 18.1 |  | 4.7 | 1.7 | 12.0 |  | 0.3 | 0.0 | 0.7 |
| **2009-2010** | 1.0 | 0.3 | 18.3 |  | 3.7 | 2.4 | 8.4 |  | 0.6 | 0.1 | 1.7 |
| **2010-2011** | 2.5 | 2.2 | 28.7 |  | 2.2 | 0.9 | 8.7 |  | 0.2 | 0.1 | 1.9 |
| **2011-2012** | 0.6 | 0.0 | 14.4 |  | 3.7 | 1.7 | 8.3 |  | 0.6 | 0.2 | 1.1 |
| **2012-2013** | 2.4 | 1.9 | 50.8 |  | 2.7 | 1.4 | 21.5 |  | 0.8 | 0.5 | 2.8 |
| **2013-2014** | 0.3 | 0.3 | 14.8 |  | 3.0 | 2.3 | 6.7 |  | 0.0 | 0.0 | 1.1 |
| **2014-2015** | 1.5 | 1.5 | 39.2 |  | 5.4 | 4.4 | 18.5 |  | 0.5 | 0.3 | 2.2 |
| **2015-2016** | 0.9 | 0.3 | 10.7 |  | 3.2 | 1.9 | 6.3 |  | 0.1 | 0.0 | 1.1 |
| **2016-2017** | 1.7 | 1.1 | 41.2 |  | 2.2 | 1.6 | 15.3 |  | 0.9 | 0.7 | 2.6 |
| **Mean** | **1.6** | **0.9** | **26.9** |  | **3.6** | **1.9** | **11.0** |  | **0.5** | **0.2** | **1.7** |
| **95% CI** | **1.0-2.3** | **0.49-1.4** | **18.3-35.5** |  | **2.6-4.5** | **1.3-2.5** | **7.4-14.5** |  | **0.26-0.80** | **0.09-0.36** | **1.3-2.2** |
| Abbreviations: CI, confidence interval. | | | | | | | | | | | |
| *Epidemiological year: August of one year through July of the following year. | | | | | | | | | | | |
| ** November 2006 until July 2007. | | | | | | | | | | | |
